# Population-Based Input Function Modeling for [^18^F]FMPEP-*d*_2_, an Inverse Agonist Radioligand for Cannabinoid CB_1_ Receptors: Validation in Clinical Studies

**DOI:** 10.1371/journal.pone.0060231

**Published:** 2013-04-05

**Authors:** Paolo Zanotti-Fregonara, Jussi Hirvonen, Chul Hyoung Lyoo, Sami S. Zoghbi, Denise Rallis-Frutos, Marilyn A. Huestis, Cheryl Morse, Victor W. Pike, Robert B. Innis

**Affiliations:** 1 Molecular Imaging Branch, National Institute of Mental Health, National Institutes of Health, Bethesda, Maryland, United States of America; 2 Chemistry and Drug Metabolism Section, National Institute on Drug Abuse, National Institutes of Health, Baltimore, Maryland, United States of America; University of Milan, Italy

## Abstract

**Background:**

Population-based input function (PBIF) may be a valid alternative to full blood sampling for quantitative PET imaging. PBIF is typically validated by comparing its quantification results with those obtained via arterial sampling. However, for PBIF to be employed in actual clinical research studies, its ability to faithfully capture the whole spectrum of results must be assessed. The present study validated a PBIF for [^18^F]FMPEP-*d*_2_, a cannabinoid CB_1_ receptor radioligand, in healthy volunteers, and also attempted to utilize PBIF to replicate three previously published clinical studies in which the input function was acquired with arterial sampling.

**Methods:**

The PBIF was first created and validated with data from 42 healthy volunteers. This PBIF was used to assess the retest variability of [^18^F]FMPEP-*d*_2_, and then to quantify CB_1_ receptors in alcoholic patients (n = 18) and chronic daily cannabis smokers (n = 29). Both groups were scanned at baseline and after 2–4 weeks of monitored drug abstinence.

**Results:**

PBIF yielded accurate results in the 42 healthy subjects (average Logan-distribution volume (*V*_T_) was 13.3±3.8 mL/cm^3^ for full sampling and 13.2±3.8 mL/cm^3^ for PBIF; R^2^ = 0.8765, p<0.0001) and test-retest results were comparable to those obtained with full sampling (variability: 16%; intraclass correlation coefficient: 0.89). PBIF accurately replicated the alcoholism study, showing a widespread ∼20% reduction of CB_1_ receptors in alcoholic subjects, without significant change after abstinence. However, a small PBIF-*V*_T_ bias of −9% was unexpectedly observed in cannabis smokers. This bias led to substantial errors, including a *V*_T_ decrease in regions that had shown no downregulation in the full input function. Simulated data showed that the original findings could only have been replicated with a PBIF bias between −6% and +4%.

**Conclusions:**

Despite being initially well validated in healthy subjects, PBIF may misrepresent clinical protocol results and be a source of variability between different studies and institutions.

## Introduction

Kinetic modeling for positron emission tomography (PET) studies often requires that tracer radioactivity concentration be measured in arterial blood. Different approaches have been tried to avoid arterial cannulation, including image-derived input function (IDIF) and population-based input function (PBIF). IDIF, which theoretically allows estimation of an individualized, non-invasive input function, measures blood tracer concentration directly from dynamic images using an anatomical blood pool, such as the heart. However, in practice, IDIF is plagued by many shortcomings. For instance, limited spatial and temporal resolution of PET cameras makes reliable estimation of radioactivity concentration in the vessels and its rapid variations over time difficult; in addition, PET cameras cannot distinguish the parent compound from its radiometabolites [1]. Moreover, IDIF can yield very biased results, depending on whether partial volume and spill-over effects are properly corrected [2], [3]. These limitations are amplified when IDIF is calculated from small vessels, such as the carotid arteries for brain studies, but are sometimes apparent even with larger blood pools [4].

PBIF modeling is based on the individual scaling of a tracer-specific input function of standard shape, and may be a more reliable alternative than IDIF. PBIF is not affected by problems linked to image quality and resolution, and radiometabolite correction can be inherently taken into account by using an average time-activity curve of parent concentrations. Moreover, because PBIF is derived from averaged values over a population, it generally shows little or no systematic error, as estimation errors of individual input functions would be normally distributed around the mean. While this may pose a problem in personalized diagnostic settings, research studies typically compare population means; thus, a larger standard deviation may be acceptable if properly accounted for.

Historically, PBIF was validated primarily for [^18^F]-FDG [5], [6], [7], [8], [9], [10], [11], [12], but recent studies showed excellent results with a wider array of tracers [13], [14], [15], [16], [17], [18], [19], [20], [21]. To our knowledge, only one reported negative results with PBIF [22]. Although publication bias is probably partly responsible, this also suggests that PBIF is a reliable technique. In the extant literature, however, PBIF is typically validated as a proof of concept method by comparing its quantification results with those obtained via arterial sampling in a group of subjects, often comprising both healthy volunteers and patients [5], [6], [7], [8], [12], [13], [14], [15], [16], [18], [19]. For PBIF to be used in research protocols, the validation procedure must be extended to assess its ability to faithfully capture the entire spectrum of results in complex clinical studies, often involving patients with different conditions.

Our laboratory developed [^18^F]FMPEP-*d*_2_, a radioligand targeting cannabinoid CB_1_ receptors [23]. [^18^F]FMPEP-*d*_2_ concentrations can be accurately measured in plasma, and this allows accurate estimation of brain distribution volume (*V*_T_) [24]. In previous studies, we assessed inter-subject and retest variability of [^18^F]FMPEP-*d*_2_ *V*_T_ values in healthy volunteers [24] and conducted two clinical studies to investigate the concentration of brain CB_1_ receptors in the brain of alcoholic subjects [25] and cannabis smokers [26]. The present study validated PBIF in healthy volunteers and also attempted to employ PBIF to replicate results from our previously published clinical studies that utilized serial arterial sampling to obtain input function.

## Subjects and Methods

Data from three previous publications involving imaging of cannabinoid CB_1_ receptors were re-analyzed. The first study (retest study) involved 8 healthy subjects (5 men and 3 women) who underwent a test-retest scan [24]. The second study (alcohol study) assessed CB_1_ receptors in 18 male patients with alcohol dependence [25]; subjects were monitored in an inpatient research unit for 2–4 weeks and CB_1_ receptors were imaged with PET and [^18^F]FMPEP-*d*_2_ at two time points: within 1 week of admission, and again after 2–4 weeks of abstinence. The third study (cannabis study) assessed CB_1_ receptors alteration in 30 males who were daily chronic cannabis smokers [26]; subjects were imaged within one day of admission onto a controlled unit, and again after 2–4 weeks of abstinence. Recruitment processes and eligibility criteria for both healthy subjects and patients are described in the original publications [24], [25], [26].

### Positron Emission Tomography and Measurement of Parent Radioligand in Arterial Plasma

Radioactivity was measured in brain with an Advance PET camera (GE Healthcare, Milwaukee, WI) over 120 minutes. The input function was measured from radial artery plasma sampling. Arterial samples were drawn at 15 seconds intervals until 2 minutes, and then at increasingly longer intervals until the end of the scan, as previously described [24], [25], [26]. Plasma time-activity curve was corrected for the fraction of unchanged radioligand by radio-high-performance liquid chromatography separation [27]. PET images were analyzed by applying a template of volumes of interest [28] as implemented in PMOD, version 3.0 (PMOD Technologies Ltd, Zurich, Switzerland) in the standard stereotactic space [29].

### Generation of the PBIF

To generate the PBIF, [^18^F]FMPEP-*d*_2_ input functions from all healthy subjects in our database were used. This comprised all healthy subjects in the three previous publications described above [24], [25], [26], as well as additional subjects recruited through July 2012. In total, data from 42 healthy subjects were available (34±10 years, 83±18 Kg, 181±9 MBq of injected activity). The National Institutes of Health (NIH) Central Nervous System Institutional Review Board approved the protocols and consent forms. Written informed consent was obtained from all subjects.

The PBIF generation process includes several steps, as described in previous publications [5], [20]: 1) each parent time-activity curve was normalized by injected activity and body weight, 2) the curves were shifted for time delay to match the average time peak, 3) each curve was fitted with a linear interpolation to the peak, followed by a tri-exponential function after the peak 4) the blood values were interpolated to the same grid of standard times (to eliminate the errors due to slightly different sampling times), 5) the normalized parent time-activity curves of all healthy subjects were averaged to obtain the final PBIF, 6) for each individual subject, the PBIF was finally individually scaled using a combination of two arterial blood samples taken at 15 and 60 minutes. This combination was selected because it yielded the highest correlation coefficient–as assessed by Pearson’s analysis–between parent activity and the total area under the curve (AUC) (Table S1).

The PBIF was first tested among healthy volunteers with a leave-one-out procedure, i.e. when testing the PBIF on a given subject, the input function of that subject was removed from the mean PBIF to avoid any bias [30]. Similarly, in the test-retest study, each retest PBIF did not include the input function from the test study of the same subject. The PBIF from all healthy subjects was then prospectively applied to the populations of alcoholics and cannabis smokers, in both test and retest scans.

### Analysis of V_T_ Data

In the original studies, kinetic modeling was performed with an unconstrained two-tissue compartmental model (2TCM) [24], [25], [26]. However, in the present work we first reanalyzed the previous studies using a Logan plot and the full arterial input function. To test the consistency and validity of Logan-derived results, the same statistical analyses used in the original studies with 2TCM were repeated with the Logan plot. As shown in Results, Logan-derived results were equivalent to those obtained using a 2TCM and were utilized as the reference standard to evaluate PBIF.

### Statistics

Statistical analyses were performed with SPSS Statistics 17.0 for Windows (Release 17.0.0, copyright SPSS Inc., 1993–2007). Retest variability (the absolute difference between the two scans divided by the mean of the two scans) and intraclass correlation coefficients (ICC) were calculated for *V*_T_ values obtained with PBIF and compared with the values obtained with the reference input function. Values of ICC were obtained by (BSMSS – WSMSS)/(BSMSS+WSMSS), where BSMSS = mean of summed squares between subjects, and WSMSS = mean of summed squares within subjects.

We sought to replicate our previous findings of region-specific and reversible downregulation of CB_1_ receptors in cannabis smokers [26], and widespread and irreversible downregulation of CB_1_ receptors in patients with alcohol dependence [25]. Downregulation at baseline was examined separately in both patient groups by applying mixed model two-way analyses of variance (ANOVA), with group status (cannabis smokers *vs.* control, or alcohol dependence *vs.* control) as a between-subjects factor and brain region as a within-subjects factor. Body mass index (BMI) entered the model as a covariate [26]. To test whether CB_1_ receptors were increased after abstinence, we applied a two-way ANOVA with time point (early *vs.* protracted abstinence) and brain region as within-subject factors. P-values smaller than 0.05 were considered statistically significant.

## Results

### Validation of PBIF in Healthy Subjects and Retest Results

**Figure 1:**
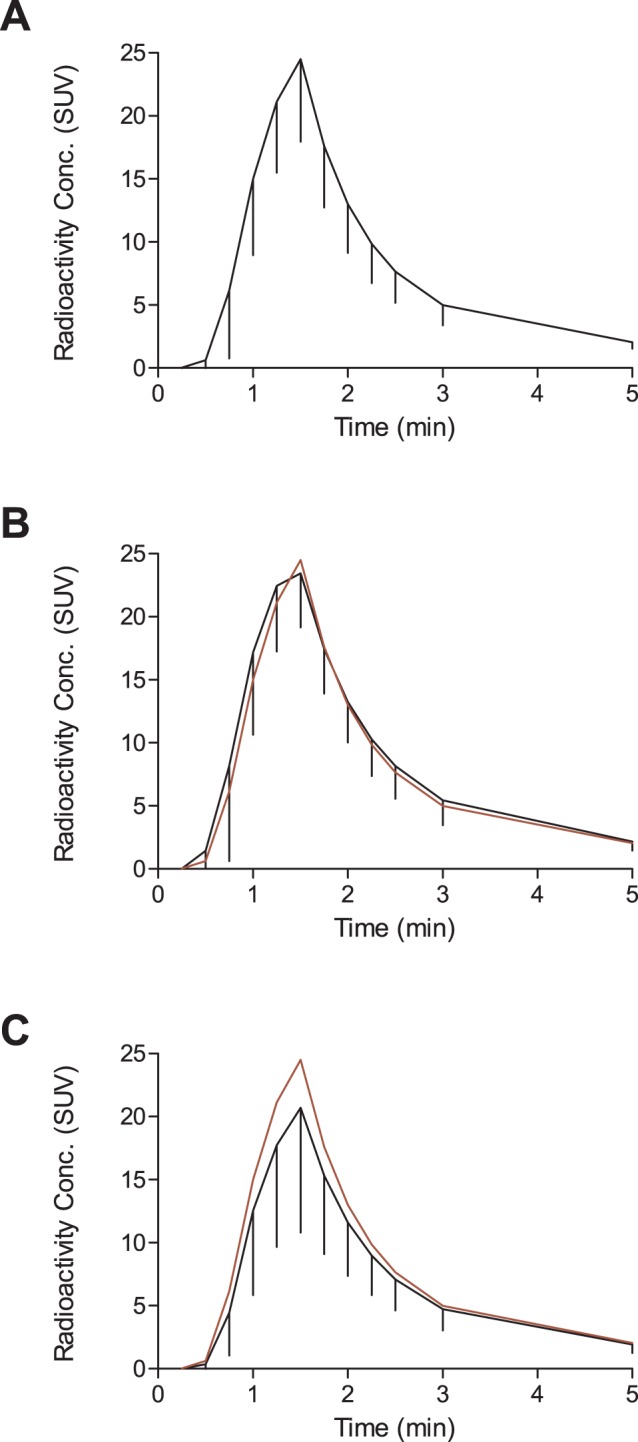
Comparison between the PBIF from healthy volunteers and the average curves in alcoholic subjects and cannabis smokers. Average PBIF, expressed in SUV, from healthy subjects (A). Error bars show standard deviations for each time point. Only the first 5 minutes are shown. After 5 minutes, all curves are virtually identical. In panel B, the black curve, with SD, represents the average input function from alcoholic subjects and the superposed red curve is the PBIF from healthy subjects. The two average population curves are very similar in shape and magnitude. However, when compared to the average curve from cannabis smokers (in black, panel C), it can be clearly seen that PBIF (in red) overestimates the peak. This will translate into a selective underestimation of Logan-*V*_T_ in the cannabis population.

Visually, PBIF curves were of very good quality (Fig. 1). The slope of the tails was very similar among the different subjects, including the transition part between the peaks and the tail. As expected, the height of the peaks showed a higher variability, with errors normally distributed around a mean value (average estimated/reference peak value: 1.00±0.21). The thickness of the peaks was also very similar among subjects. Kinetic modeling showed that the Logan-*V*_T_ (mean of all regions) in the 42 healthy subjects was 13.3±3.8 mL/cm^3^ for Logan blood and 13.2±3.8 mL/cm^3^ for Logan PBIF (*V*_T_ ratio = 1.00±0.12). The correlation between the two *V*_T_ estimates was good (R^2^ = 0.8765, p<0.0001). Most individual subjects showed small *V*_T_ estimation errors (<10%).

In the original test-retest study done with 2TCM, the retest variability was 14% and the ICC value was 0.89 [24]. When the Logan plot and the full input function were employed in the present study, retest variability was slightly higher (16%), although the ICC value was similar (0.88). This slightly lower mean 2TCM retest variability appeared to be due to lower variability in subcortical regions, such as the pons and the white matter; cortical regions, which are more relevant for CB_1_ analysis, displayed similar values (Table 1).

**Table 1:** Retest variability for 2TCM, Logan with full input, and Logan with PBIF, respectively.

	2TCM full input		Logan full input		Logan PBIF	
Region	Retest Variability (%)	ICC	Retest Variability (%)	ICC	Retest Variability (%)	ICC
Prefrontal	16	0.91	17	0.89	14	0.89
Occipital	13	0.90	15	0.90	17	0.89
Hippocampus	15	0.86	18	0.85	14	0.88
Putamen	17	0.88	18	0.87	17	0.86
Thalamus	15	0.89	17	0.87	17	0.90
Cerebellum	15	0.85	15	0.86	14	0.88
Pons	9	0.94	13	0.93	17	0.92
White Matter	11	0.88	16	0.87	17	0.89

In the present study, results with Logan and the PBIF (variability: 16%; ICC = 0.89) were nearly identical to those obtained with Logan and the full input function. *V*_T_ values obtained with 2TCM, Logan with full input and Logan with PBIF were strongly correlated (scatter plots are shown in Figure S1).

### Alcohol Study

In the alcohol study, the Logan full input model showed a widespread decrease in *V*_T_ of [^18^F]FMPEP-*d*_2_ among patients with alcohol dependence compared with healthy subjects (main effect of group: F = 9.06, p = 0.005) that, although found in all brain regions, varied in magnitude across regions (group×region interaction: F = 6.42, p = 0.002). This change was similar in magnitude to that observed with compartment modeling in the original study [25]. After 2–4 weeks of abstinence, Logan-*V*_T_ did not change from baseline in any region (main effect of repetition: F = 0.69, p = 0.417; repetition×region interaction: F = 1.08, p = 0.362), and percent change was similar to that observed with the compartment model [25].

**Figure 2:**
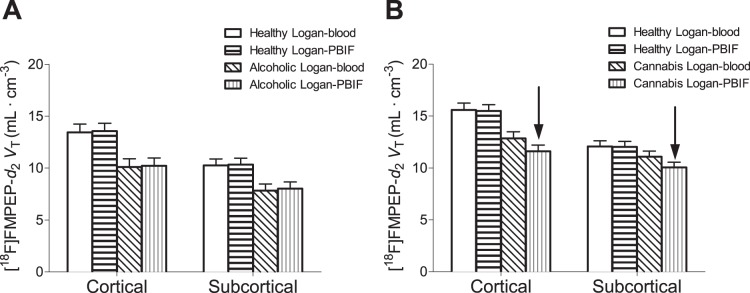
Comparison of Logan-*V*_T_ results in cortical and subcortical regions for healthy volunteers, alcoholic subjects and cannabis smokers, obtained with full input and the PBIF. Results at baseline in alcoholic subjects (A) and cannabis smokers (B), compared to healthy controls for both full input and PBIF. PBIF provided accurate Logan-*V*_T_ values in healthy subjects and alcoholic patients and closely replicated previous findings of widespread downregulation. In cannabis smokers, PBIF underestimated Logan-*V*_T_ (arrows); the results suggest the erroneous conclusion that downregulation also occurred in subcortical regions.

In the present study, PBIF accurately estimated *V*_T_ values among alcoholic subjects. The Logan-*V*_T_ was 9.4±2.6 mL/cm^3^ for Logan blood and 9.5±2.6 mL/cm^3^ for Logan PBIF (*V*_T_ ratio = 1.02±0.14; R^2^ = 0.7774, p<0.0001). PBIF accurately replicated the widespread decrease in *V*_T_ in alcoholic subjects both in terms of magnitude and statistical significance of change (main effect of group: F = 8.99, p = 0.005; group×region interaction: F = 8.32, p = 0.0002) (Fig. 2A). After abstinence, PBIF modeling showed a similar lack of change in *V*_T_ (main effect of repetition: F = 1.86, p = 0.191; repetition×region interaction: F = 1.27, p = 0.294) (Table 2).

**Table 2:** Results from the alcohol and cannabis studies using PBIF.

	Full input(2TCM and Logan)	PBIF(Logan)
Cannabis baseline	Regionally selective downregulation	**Overestimation of downregulation,** **non-regionally selective**
Cannabis abstinence	Regionally selective increase	Regionally selective increase
Alcohol baseline	Widespread downregulation	Widespread downregulation
Alcohol abstinence	No change	No change

Bold text underscores differences between PBIF and full sampling modeling.

### Cannabis Study

The Logan model with full arterial input function obtained results similar to those found with 2TCM [26]. A regionally specific decrease in *V*_T_ of [^18^F]FMPEP-*d*_2_ was found in cannabis smokers compared with healthy subjects (group×region interaction: F = 10.0, p = 0.00004). Compared to the original paper which included 30 cannabis smokers [26], one was removed from the analysis because the Logan plot did not provide a correct linearization. As with 2TCM, *V*_T_ was about 20% lower in neocortical and limbic regions, but not in other brain regions (such as cerebellum and basal ganglia) [26]. After about 4 weeks sustained cannabis abstinence, *V*_T_ increased specifically in those brain regions that had shown decreased *V*_T_ at baseline (repetition×region interaction: F = 4.58, p = 0.006).

**Figure 3:**
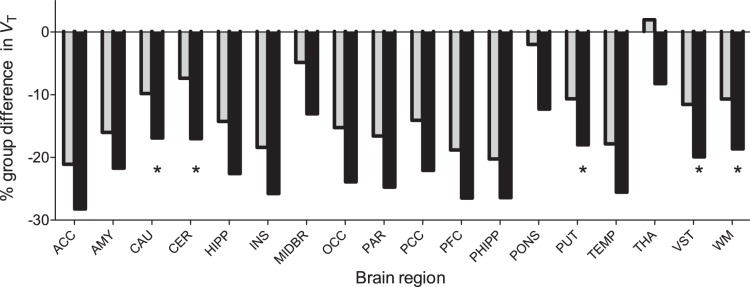
Regional percent differences in [^18^F]FMPEP-*d*_2_ *V*_T_ between healthy subjects and chronic cannabis smokers. *V*_T_ values are estimated by the Logan method with full arterial input curve (gray bars) and with PBIF (black bars). Compared with full arterial input modeling, PBIF underestimated *V*_T_ by about 9% in the cannabis group and erroneously showed statistically significant downregulation in subcortical regions that did not differ in either compartmental kinetic modeling or Logan full arterial input modeling (asterisks).

The Logan-*V*_T_ obtained with PBIF showed however a small bias of about −9% as compared to the Logan-*V*_T_ obtained with the full input function (12.7±3.4 mL/cm^3^ for Logan blood and 11.5±3.1 mL/cm^3^ for Logan PBIF; *V*_T_ ratio = 0.91±0.13; R^2^ = 0.7057, p<0.0001) (Fig. 1). Because of this bias, PBIF modeling found a larger decrease in *V*_T_ in cannabis smokers (−27% in the prefrontal cortex compared with −19% using full input), and the group×region interaction was statistically highly significant (F = 17.1, p = 0.00000001) (Fig. 2B). However, *V*_T_ was also decreased in regions that had shown no downregulation at baseline with the full input function, namely, caudate, putamen, ventral striatum, cerebellum, and white matter (Fig. 3). After abstinence, PBIF modeling showed similarly significant region-specific changes (repetition×region interaction: F = 4.96, p = 0.011), although percent change was higher than that obtained with the Logan full input model. Notably, PBIF-*V*_T_ recovered after abstinence only in neocortical and limbic brain regions, because other regions (such as white matter) showed no physiological change between baseline and abstinence conditions.

Because the bias of −9% was sufficient to cause substantial errors in the interpretation of the regional data from the cannabis study, we created datasets with different degrees of simulated bias (1 percent unit increments from −8% to +9%) to find the tolerable range of bias that would allow the exact replication of the results. In practice, starting from the actual PBIF-*V*_T_ values, we created simulated sets of *V*_T_ values whose means equal the target bias and the coefficient of variation is constant and equal to that of the real PBIF-*V*_T_ values. The simulated data showed that significant deviations from the original findings of the study appeared with a bias lower than −6% or higher than +4%. Negative bias resulted in false positive findings in a number of brain regions. With a −6% bias, basal ganglia (caudate and ventral striatum) appeared to be significantly downregulated, a finding that is inconsistent not only with our previous kinetic modeling study [26], but also with animal studies showing resistance of basal ganglia CB_1_ receptors to chronic agonist-induced downregulation [31]. Conversely, positive bias reduced statistical power and caused false negative findings. For example, a bias of +4% was sufficient to cause lack of significant downregulation in 6 out of 10 cortical and limbic brain regions, despite the fact that downregulation was found in all regions with full arterial input modeling. Notably, if the −9% PBIF *V*_T_ bias would have occurred in the other direction (i.e. +9%), the decrease would not have been statistically significant in any of the individual brain regions (although the study still would have demonstrated a decrease in *V*_T_ -group×region interaction: F = 4.17, p = 0.014); furthermore, no significant recovery of receptor downregulation after abstinence would have been observed (repetition×region interaction: F = 2.16, p = 0.097).

## Discussion

This study assessed whether PBIF can reliably replicate actual clinical protocols by comparing PBIF results to those obtained with a full input function.

Before analyzing PBIF data, we first replicated the previous studies using a Logan plot and the full arterial input function. This was necessary to have data that were directly comparable to those acquired using the Logan plot and the PBIF. In fact, compartmental modeling is not well suited to PBIF, because *V*_T_ is derived by combining individual rate constants, which are sensitive to the shape of the input function. PBIF cannot accurately reproduce the shape of the individual input functions, especially during the early rapid phase. In contrast, the Logan graphical plot relies on the AUC of the input function [32], which can be correctly estimated even with a differently shaped curve. Consequently, Logan is preferable to compartmental modeling when less invasive methods are used to obtain the arterial input function (e.g., PBIF or IDIF). As expected, Logan-derived results were equivalent to those obtained using a 2TCM and were utilized as the reference standard to evaluate PBIF.

In a large number of healthy subjects, both at baseline and in a test-retest setting, PBIF for [^18^F]FMPEP-*d*_2_ gave reliable results. Moreover, the PBIF obtained from healthy subjects also closely replicated results from the alcohol study, showing a widespread decrease in *V*_T_ in patients with alcohol dependence, both in terms of magnitude and statistical significance of change. After 2–4 weeks of abstinence, kinetic modeling with both full input and PBIF showed a similar persistent downregulation of CB_1_ receptors.

However, despite these initial good results, PBIF-derived results in cannabis smokers had an unexpected small bias of about −9%, and therefore differed in some important ways from the reference values. Had PBIF been used exclusively, we would not have missed the main finding of the study, i.e. that cannabis smoking is associated with a downregulation of CB_1_ receptors in the brain that is reversible after abstinence. However, we would have incorrectly concluded that receptor downregulation occurred in all brain regions non-selectively, which is inconsistent with the known physiology of CB1 receptors [26] [31]. Notably, after abstinence PBIF-*V*_T_ recovered only in neocortical and limbic brain regions, because other regions (such as white matter) showed no physiological change between baseline and abstinence conditions. This further supports the hypothesis that the downregulation seen at baseline in those regions with PBIF was erroneous.

Simulated data showed that the range of bias that would have allowed us to substantially replicate the original findings of the cannabis study was between −6% and +4%. This quite narrow margin of error is obviously specific to this population and this study. Nevertheless, it is important to underscore that, for any new study, it cannot be known beforehand what an acceptable bias would be. Notably, small biases of about 10%–even at initial validation–are not uncommon with PBIF [7], [19].

With PBIF, estimated errors of *V*_T_ are supposed to be normally distributed around the mean. However, out of 29 cannabis smokers, 27 showed underestimated *V*_T_ values. Therefore, the bias was not due to some individual outlying values, but to a systematic difference uniform throughout the group. The mechanistic explanation of this *V*_T_ underestimation is a slightly different shape in the input function of cannabis smokers (Fig. 1). Indeed, the arterial input functions of cannabis smokers, although they are not statistically separable with a t-test, have a lower peak compared to both healthy subjects and alcoholics (Table 3). The AUC, expressed in SUV, of the first 5 minutes (AUC_0–5_) in cannabis smokers was 33.4, while it was 38.1 in healthy controls and 40.5 in alcoholic subjects. The rest of the input function (AUC_5–120_) was very similar among the three groups (32.6, 32.7 and 32.2, respectively). Indeed, the blood sample values of cannabis subjects used for scaling the PBIF (0.79±0.19 SUV at 15 minutes and 0.15±0.04 SUV at 60 minutes) were similar to those of healthy subjects (0.75±0.23 SUV and 0.15±0.06 SUV, respectively).

**Table 3:** Areas under the curve, expressed in SUV, of the peak area (first 5 minutes) and the rest of the arterial input function in the three populations of subjects.

	Healthy (n = 42)	Alcohol (n = 18)	Cannabis (n = 29)
AUC_0–5_	38.1±9.6	40.5±9.0	33.4±12.4
AUC_5–120_	32.7±9.9	32.2±7.5	32.6±6.9
AUC_TOTAL_	70.8±18.5	72.7±15.4	66.0±16.1

We have no clear explanation for the blunted peak observed in the cannabis subjects. All groups underwent the same procedure with regard to radioligand preparation and measurement, and were injected with an automated pump over the same time span of one minute, which ensures a reproducible shape of the injected bolus. Fifteen second-sampling was used during the first two minutes to sample the input function. A continuous blood sampling device may allow a better definition of the shape of the peaks and should preferably be used for future studies. However that would be an unlikely explanation for our findings, because all input functions were acquired with the same time frame. Cannabis smokers had a lower BMI (24±4 vs. 27±5 kg/m^2^), more tobacco use, and higher alcohol consumption [26] (which should not have significantly contributed to the bias, given that we accurately replicated the study in alcoholic subjects). Hypothetical gender-related differences are unlikely to be the cause, because 86 of the 89 subjects in the present study were males. Also, the fraction of free radioligand in plasma of cannabis smokers (0.42±0.2%) was similar to that of healthy controls (0.40±0.2%) [26]. Notably, cannabis smokers do not display a different metabolism rate of the radiotracer, since the AUC of the tail of the input function is very similar to that of normal subjects and alcoholics (and very little metabolism occurs in the first five minutes after injection). One speculative explanation is that cannabis smokers may have a slightly slower metabolism than healthy subjects, which would explain a parent concentration at late time-points similar to that of the other groups, despite an initial lower peak.

However, independent of the underlying pathophysiological reason in this specific case, the message of this paper is that PBIF can be very sensitive to quantitatively minor and unforeseeable differences that may occur when transposing a given PBIF to different populations of subjects. Moreover, all populations studied in the present work were composed of age-matched, young persons, free of organic disease. It is conceivable that bias estimations found between comparable groups of healthy individuals would be amplified if one group was composed of patients with systemic, cardiovascular or neoplastic diseases.

It is certainly conceivable that better results would have been obtained by using different PBIFs derived from each population (indeed, a PBIF from the cannabis smokers of the present study predictably shows a lower peak than the PBIF from healthy subjects). However, this approach would be unfeasible in clinical practice, because a PBIF for each specific population of patients to be studied is usually not available. Moreover, even a PBIF derived from a specific group of patients may not be prospectively applicable to patients with a different degree of disease.

A 1994 study by Takikawa and colleagues provides a rare example where the performance of PBIF was tested against the full input in both healthy subjects and patients, and results were correlated with clinical findings [15]. Although an [^18^F]-DOPA PBIF still correctly discriminated between healthy subjects and patients with Parkinson’s disease, it predicted quantitative severity ratings less accurately. Striatal *K*_i_ correlated significantly with the Unified Parkinson’s Disease Rating Score using the reference input function, but this correlation was lost when PBIF was employed.

In summary, care should be taken when using alternative techniques to measure input function. Despite initially good validation in healthy subjects, errors may unexpectedly arise in actual clinical protocols. These errors, in turn, may lead to a misinterpretation of results and be a source of variability between different studies and institutions. Indeed, these erroneous results may have important consequences for understanding basic pathophysiology or pharmacological effectiveness of drugs. Discrepant results are unfortunately not rare in molecular imaging studies [33], [34]. While clinical variability is surely partly responsible, methodological issues–including the choice of input function–are likely to play an important role [34], [35]. Therefore, whenever possible, the input function should be obtained with the gold standard of serial arterial sampling with individual correction for radiometabolites.

## Supporting Information

Figure S1 **Scatter plots of 2TCM/Logan with full input, 2TCM/Logan with PBIF and Logan with full input/Logan with PBIF.** Values for the test scans are reported in the left column and those of the retest scans in right column. For each one of the test-retest subjects, a single *V*_T_ value (whole-brain region) has been used. A strong correlation exists between all datasets.
(EPS)

Table S1 **Correlation coefficients for the area-under-the-curve of the plasma time-activity curve (AUC) using the measured radioligand concentrations.**
(DOCX)
