# Supplementary material for: Population-Based Input Function Modeling for [18F]FMPEP-d 2, an Inverse Agonist Radioligand for Cannabinoid CB1 Receptors: Validation in Clinical Studies
Source: PLoS One. 2013 Apr 5;8(4):e60231. doi: 10.1371/journal.pone.0060231 (PMC3618181; doi:10.1371/journal.pone.0060231)
Supplement: Table S1 — Correlation coefficients for the area-under-the-curve of the plasma time-activity curve (AUC) using the measured radioligand concentrations. (DOCX) [file pone.0060231.s002.docx]

Table S1. Correlation coefficients for the area-under-the-curve of the plasma time-activity curve (AUC) using the measured radioligand concentrations

Time of first plasma concentration (min)

| Time of second plasma concentration (min) |  | 1.5 | 3 | 5 | 8 | 10 | 15 | 20 | 30 | 45 | 60 | 75 | 90 | 105 | 120 |
| --- | --- | --- | --- | --- | --- | --- | --- | --- | --- | --- | --- | --- | --- | --- | --- |
|  | 1.5 | *0.857* | 0.877 | 0.870 | 0.867 | 0.868 | 0.867 | 0.866 | 0.863 | 0.861 | 0.859 | 0.859 | 0.858 | 0.858 | 0.858 |
|  | 3 |  | *0.690* | 0.766 | 0.782 | 0.773 | 0.754 | 0.743 | 0.731 | 0.720 | 0.712 | 0.706 | 0.702 | 0.698 | 0.696 |
|  | 5 |  |  | *0.843* | 0.871 | 0.887 | 0.907 | 0.912 | 0.904 | 0.886 | 0.873 | 0.866 | 0.862 | 0.859 | 0.857 |
|  | 8 |  |  |  | *0.867* | 0.880 | 0.904 | 0.916 | 0.919 | 0.908 | 0.899 | 0.893 | 0.890 | 0.887 | 0.886 |
|  | 10 |  |  |  |  | *0.889* | 0.907 | 0.917 | 0.927 | 0.927 | 0.922 | 0.918 | 0.914 | 0.911 | 0.908 |
|  | 15 |  |  |  |  |  | *0.905* | 0.905 | 0.914 | 0.930 | **0.937** | 0.936 | 0.932 | 0.927 | 0.921 |
|  | 20 |  |  |  |  |  |  | *0.892* | 0.889 | 0.906 | 0.918 | 0.921 | 0.918 | 0.912 | 0.904 |
|  | 30 |  |  |  |  |  |  |  | *0.863* | 0.864 | 0.875 | 0.881 | 0.882 | 0.877 | 0.871 |
|  | 45 |  |  |  |  |  |  |  |  | *0.842* | 0.842 | 0.846 | 0.849 | 0.848 | 0.846 |
|  | 60 |  |  |  |  |  |  |  |  |  | *0.831* | 0.829 | 0.828 | 0.827 | 0.824 |
|  | 75 |  |  |  |  |  |  |  |  |  |  | *0.819* | 0.810 | 0.801 | 0.791 |
|  | 90 |  |  |  |  |  |  |  |  |  |  |  | *0.790* | 0.768 | 0.743 |
|  | 105 |  |  |  |  |  |  |  |  |  |  |  |  | *0.727* | 0.684 |
|  | 120 |  |  |  |  |  |  |  |  |  |  |  |  |  | *0.903* |

The values along the diagonal (in italics) are the correlation coefficients between the activities of individual time points with the AUCs. The other values are the correlation coefficients between the AUC and the mean activities of two individual samples, covering all possible combinations. The highest correlation value (in bold) was found using two blood samples at 15 and 60 min.
